# Glycemic Index of Gluten-Free Bread and Their Main Ingredients: A Systematic Review and Meta-Analysis

**DOI:** 10.3390/foods10030506

**Published:** 2021-02-27

**Authors:** Bernardo Romão, Ana Luísa Falcomer, Gabriela Palos, Sandra Cavalcante, Raquel Braz Assunção Botelho, Eduardo Yoshio Nakano, António Raposo, Faiyaz Shakeel, Sultan Alshehri, Wael A. Mahdi, Renata Puppin Zandonadi

**Affiliations:** 1 Department of Nutrition, Faculty of Health Sciences, Campus Universitário Darcy Ribeiro, University of Brasilia, Brasilia, DF 70910-900, Brazil; bernardolima156@gmail.com (B.R.); anafalcomer@gmail.com (A.L.F.); gabipalos@gmail.com (G.P.); sandrauck@gmail.com (S.C.); raquelbotelho@unb.br (R.B.A.B.); 2 Department of Statistics, University of Brasilia, Brasilia, DF 70910-900, Brazil; eynakano@gmail.com; 3 CBIOS (Research Center for Biosciences and Health Technologies), Universidade Lusófona de Humanidades Tecnologias, Campo Grande 376, 1749-024 Lisboa, Portugal; 4 Department of Pharmaceutics, College of Pharmacy, King Saud University, Riyadh 11451, Saudi Arabia; faiyazs@fastmail.fm (F.S.); salshehri1@ksu.edu.sa (S.A.); wmahdi@ksu.edu.sa (W.A.M.); 5 Department of Pharmaceutical Sciences, College of Pharmacy, Almaarefa University, Riyadh 11597, Saudi Arabia

**Keywords:** gluten-free bread, glycemic index, ingredients

## Abstract

This study aimed to perform a systematic review and meta-analysis of the glycemic index (GI) of gluten-free bread (GFB) and its main ingredients. The systematic review followed PRISMA guidelines, using seven electronic databases (PubMed, EMBASE, Scopus, Science Direct, Web of Science, gray literature research with Google Scholar, and patents with Google Patent tool), from inception to November 2020. Eighteen studies met the inclusion criteria evaluating 132 GFB samples. Five articles tested GI *in vivo*, eleven *in vitro*; and two studies tested both methods. The analysis showed that 60.7% (95% CI: 40.2–78.1%) of the samples presented high glycemic indexes, evidencing a high glycemic profile for GFB. Only 18.2% (95% CI: 11.7–27.2%) of the bread samples presented in the studies were classified as a low GI. Meta-analysis presented moderate/low heterogenicity between studies (*I*^2^ = 61% and <1% for both high and low GIs) and reinforced the proportion of high GIs. Lower GIs were found in formulations based on *Colocasia esculenta* flour or enriched with fiber, yogurt and curd cheese, sourdough, psyllium, hydrocolloids, enzymes, fructans, and resistant starch, highlighting the efficacy of these ingredients to lower GFBs’ GI. GFB tends to present high GI, impacting the development of chronic diseases when consumed.

## 1. Introduction

The gluten-free diet (GFD) has become more popular since it is the only treatment for individuals with gluten-related disorders (GRD) [1,2]. Despite the benefits of gluten-exclusion for those who need to follow strict GFD, there are divergences regarding the nutritional quality and unhealthy effects of gluten-free products [3]. Unbalanced GFD is correlated to an increase in chronic diseases, highlighting the importance of improving the nutritional quality of gluten-free (GF) products [1]. Gluten-free starches and flours are traditionally low in fiber, micronutrients, protein, and, usually, present a higher glycemic index (GI) [3,4]. The GI is an essential tool in the evaluation of the nutritional quality of food since high or upper-limit moderate GI is related to the increasing prevalence of diabetes, being overweight, and cardiovascular diseases [5,6]. Therefore dietary guidelines suggest a diet with low GI foods (<55) to prevent chronic diseases [6,7,8].

Among foods with a high GI, bread is one of the most popular items in the consumer’s shopping basket [9], reaching the worldwide average consumption of 18 kg/year per capita [10,11]. Among gluten-free products, bread is the most desired product by people with a GRD. However, challenges regarding gluten replacement often lead to poor sensorial and technological quality [4,12,13]. Aiming to meet the need for gluten-free bread and the desires of people with a GRD, studies have been searching for a Gluten-Free Bread (GFB) with similar quality aspects to bread containing gluten [14]. However, most of these products’ present poor nutritional quality (highly starchy and fatty, and low in protein, fiber, and micronutrients) to compensate for the gluten absence and to achieve the sensorial and technological quality.

Given the growing popularity of gluten-free products, it is necessary to best inform individuals with a GRD regarding the health implications of gluten-free food consumption, mainly of the risk for chronic diseases [15]. Therefore, this study aimed to perform a systematic review of the glycemic index of gluten-free bread and the main ingredients used in their formulations following PRISMA guidelines. This information will potentially allow health professionals and consumers to guide their diet to avoid health impairments.

## 2. Materials and Methods

In November 2020, we performed a systematic review to evaluate and compare the characteristics of GFBs’ GI and its main ingredients. The increasing demand for gluten-free products, primarily GF bread with a good nutritional profile and sensory quality, justify the importance and the need to evaluate the glycemic index of gluten-free bread and the main ingredients used in their formulations. For scientific rigor, this systematic review was reported according to the Preferred Reporting Items for Systematic Reviews and Meta-Analyses (PRISMA) Checklist [16], as described in the Supplementary File S1 and Guidance of the European Food Safety Authority [17]. The PICOS criteria used to define the research questions were: Participants: Human subjects or *in vitro* human digestion simulation; Intervention: Glycemic index gauging; Comparison: Glycemic index of glucose or white bread; Outcome measure: Glycemic impact; Type of studies included: Scientific manuscripts and patents (Supplementary File S1).

### 2.1. Protocol and Registration

The protocol study was not recorded in PROSPERO (an international database of prospectively registered systematic reviews in health and social care, welfare, public health, education, crime, justice, and international development, where there is a health-related outcome) since this platform is not focused to reviews with food as the main subject.

### 2.2. Eligibility Criteria

The workgroup determined the inclusion and exclusion criteria for the literature search and identified search terms for each research question, as described below.

#### 2.2.1. Inclusion Criteria

Experimental studies regarding GFB’s GI determination of commercial or developed GFB formulations based on different types of gluten-free starches and flours or gluten replacements were included. We also explored their respective influence on the GI. 

#### 2.2.2. Exclusion Criteria

Exclusion criteria: reviews, letters, conference summaries, case reports, short communications, and books; studies of other GF products.

### 2.3. Information Sources 

We developed detailed individual search strategies for each of the following databases: PubMed, EMBASE, Scopus, Science Direct, Web of Science, and gray literature (Google Scholar). We searched registers of patents through the six mentioned databases and the Google Patent tool. The last search was performed on 18 November 2020. Additionally, we examined the reference lists of articles selected for full-text reading for possible relevant studies that were not retrieved by the search during the electronic search on databases.

### 2.4. Search Strategy 

A comprehensive literature search using the eligibility criteria defined by the workgroup using the mentioned databases was conducted. During the question development (Supplementary File S1), the workgroup identified key terms and outcomes. These terms, along with identified outcomes, were used to conduct the literature search. The used key terms were combined or isolated in all databases: glycemic index; glycemic impact; glycemic; index; blood glucose; blood sugar; glycemic response; postprandial glycemia; postprandial blood glucose response; postprandial blood glucose; postprandial glucose; glycemic curve; hydrolysis curve; starch hydrolysis; starch digestion; starch absorption; nutritional quality; nutritional balance; gluten-free diet; gluten-free; gluten-free products; bread; gluten-free bread. The Rayyan software (Qatar Computing Research Institute-QCRI) was used to assist in selecting and deleting duplicate articles, and all references were managed using the Endnote desktop software. Each step was systematic, reproducible, and clearly documented for transparency (Supplementary File S1).

### 2.5. Study Selection

**Figure 1:**
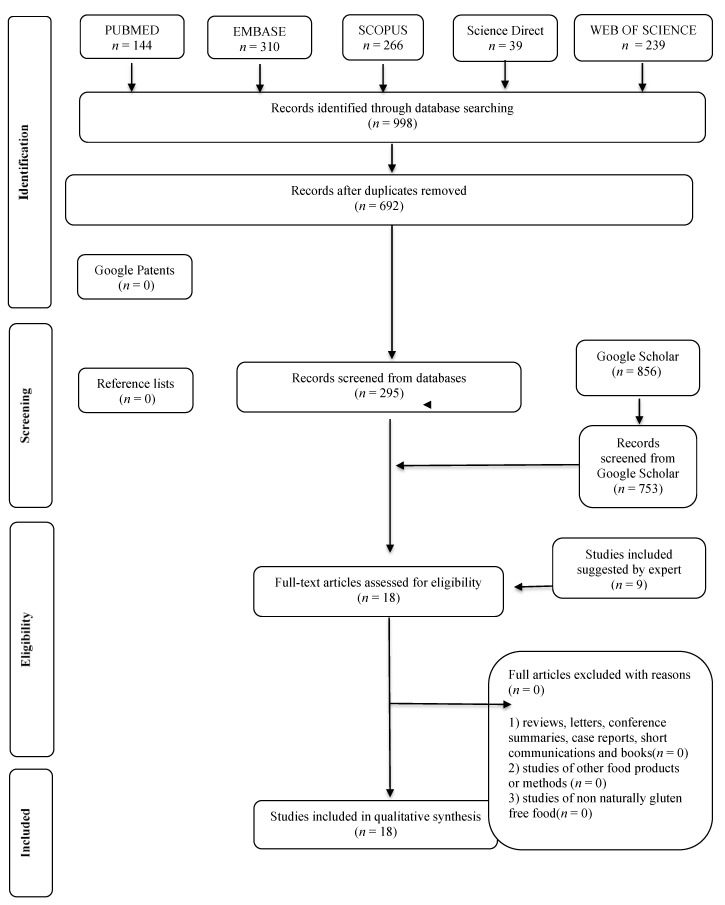
Flow Diagram of Literature Search and Selection Criteria.

We conducted the studies’ screening in 2 phases. In phase 1, two reviewers (GP, SC) independently reviewed the titles and abstracts of all references identified from databases. Articles that did not meet the eligibility criteria were discarded. In phase 2, the same reviewers (GP, SC) applied the eligibility criteria to the full texts of the selected articles. In cases of disagreement, in both phases, the two reviewers discussed the issue until a consensus. In situations where there was no consensus, a third reviewer (BR) made the final decision. The final selection was always based on the complete text of the publication. The ALF examiner critically evaluated the list of references of the selected studies. Two reviewers (GP, SC) extracted data. The third reviewer (BR) and the expert (RPZ) added additional studies. Table S1 describes the adopted quality criteria, and the flow diagram of the literature search and selection criteria is shown in Figure 1. 

### 2.6. Data Collection Process

The following characteristics were selected from the articles: authors and year of publication, country of the study, study aim, study outline, starch sample/blood sample analysis method, type of flour/starch base, production (if available in local markets or developed by the study’s authors), enrichment, *in vivo*/*in vitro*, number of samples/subjects tested, use of control samples and method to determine GI. Calibration exercises were performed before starting the review to ensure consistency among reviewers. Reviewers solved disagreements by discussion, and the third reviewer (BR) adjudicated unresolved disagreements. 

All literature searches and results were documented in the search plan (which included the study question, month and year of the literature review, inclusion and exclusion criteria, and search terms). As mentioned, the report was based on the PRISMA flowchart. After completion of the data extraction and quality appraisal process, data were synthesized by three researchers (GP, SC, BR) using a standardized table containing information about reference; country; aim; bread starch sample/blood sample analysis method, type of flour/starch base, enrichment (yes/no and which ingredient used), *in vivo*/*in vitro*, number of samples/subjects tested (if triplicates/duplicates, or if healthy subjects or not), use of control samples (yes/no) and method to determine GI, since synthesizing evidence summaries involves combining relevant and valid information [18]. The Wordcloud® tool was used to highlight the most mentioned ingredients and the studies used as gluten-replacements or starch sources. 

### 2.7. Risk of Bias 

Once the studies that met the systematic review’s inclusion criteria were identified, each study was carefully assessed for methodologic quality. Additionally, outcomes of interest were extracted, the evidence was summarized, and the strength of evidence was assessed. Therefore, the critical appraisal (risk of bias) for each study was conducted. The quality criteria were synthesized using the Meta-analysis of Statistics Assessment and Review Instrument (MASTARI) protocol [19] to evaluate the articles’ risk of bias. The bias risk assessment instrument included nine questions. The risk of bias was classified as “high” when the study reached up to 49% of the answer “yes”, “moderate” when the study reached 50% to 69% of the answer “yes”, and “low” when the study presented more than 70% of the answer “yes” (Tables S2 and S3).

### 2.8. Meta-Analysis 

Since the population-based studies are likely heterogeneous, a random-effect model was considered [19]. Estimates of percentage (of bread with high and low GIs) were transformed using the logit transformation to fit confidence intervals of estimates equals (or close) to 0% and 100%. The *I*^2^ statistic was used to evaluate the proportion of heterogeneity among the studies. An *I*^2^ value between 50% and 75% was considered moderate heterogeneity, and a value greater than 75% was considered as high [19]. The Forest Plots showed the heterogeneity of the studies. The *Metaphor* package *R*-program performed the meta-analysis.

## 3. Results 

**Figure 2:**
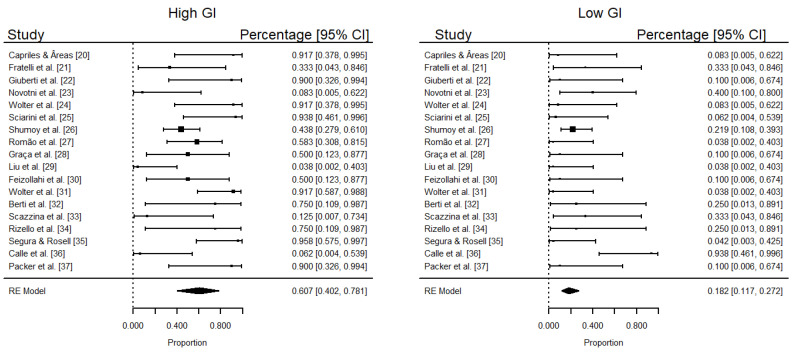
Percentage of bread with high and low glycemic indexes in each study. Percentages and confidence intervals estimated by logit transformation for proportions. Diamonds represent the pooled estimates (95% CI) obtained by Random-Effect Model. Ref [20,21,22,23,24,25,26,27,28,29,30,31,32,33,34,35,36,37].

In all searched electronic databases, we identified 364 articles. We did not find patent registers of GFB that included a GI analysis. In Phase 1, we selected 18 articles for their potential interest in Phase 2. Specialists did not suggest reading other articles. Thereby, we ended with 18 articles for a complete reading. From these, all met the eligibility criteria, and all the included studies were published between 2000 and 2020. The 18 selected studies resulted in a pooled sample size of 132 GFB. A summary of descriptive characteristics and outcomes of interest in the included studies is available at Table 1. The pooled analysis estimates an overall percentage of 60.7% (95% CI: 40.2–78.1%) of high GIs (≥70) (Table 2, Figure 2). Only 18.2% (95% CI: 11.7–27.2%) of the bread was classified as low GI (≤55), evidencing a high glycemic profile for GFB. According to the meta-analysis, as expected, there is a moderate/low level of heterogeneity between studies (*I*^2^ = 61% and <1% for high and low GI outcomes, respectively) (Figure 2). Additionally, a high proportion of high GIs in GFB was evidenced by the meta-analysis (Figure 2). From the GFB samples classified as low GI, ingredients such as *Colocasia esculenta* flour, fiber, psyllium, inulin-type fructans, sourdough, and resistant starch were used [20,21,22,23,24] (Table 1 and Table 2).

### Studies General Characteristics

The elected studies took place in nine different countries: Argentina [25], Belgium [26], Brazil [20,21,27], Portugal [28], Croatia [23], China [29], Iran [30], Ireland [24,31], Italy [22,32,33,34], Spain [35,36], and the United Kingdom [37] (Table 1). GI’s of the samples were determined with two different methods, *in vitro*, representing 61.11% (*n* = 11) of the total selected studies [22,24,25,28,29,30,31,34,35,36] and *in vivo*, representing 27.77% (*n*= 5) [21,23,27,33,37] (Table 1). Two of the studies (11.1%) determined the GIs of GFB samples by both methods [20,32].

From all studies, there were 22.2 (*n* = 4) performed analysis on GFB brands available on local markets [28,32,34,36](Table 1). In comparison, 77.77% (*n* = 14) developed their GFB samples based on different starches/flours, predominantly rice, potato, and cassava, and stabilizing agents, such as resistant starch, psyllium, sourdough, and various hydrocolloids [23,25,26,27,29,31,32,33,36,37]. A summary of descriptive characteristics and outcomes of interest in the included studies is available in Table 1. 

## 4. Discussion

The GI in the food label is being discussed in some countries [46,47,48,49]. In this sense, labeling of foods for the GI could inform consumers how to choose carbohydrate-containing foods based on their potential physiological effects [46]. There is good evidence that foods with low GI improve overall blood glucose, reduce body serum lipids, improve insulin sensitivity reducing the risk for type 2 diabetes development and cardiovascular disease [5,6,7,8,46]. However, there are no clear directions regarding standardized methodology as the reference, total available carbohydrate of the tested food, number, characteristics, and acknowledgment of variations between experimental subjects, capillary versus venous blood samples, and analytical method (*in vitro* or *in vivo*) [50]. Therefore, some countries’ regulatory agencies did not adopt the GI in food labels [47,51,52], showing the importance of studies regarding the GI on foods. To our knowledge, only South Africa, Canada, Australia, and New Zealand presented provisions for GI claims in their regulations [47,53], and other countries are still evaluating the regulation of the health claims related to the GI food labeling [46,47,48,54].

Some authors mention that gluten-free bread presents high GI and the use of ingredients/additives rich, mainly in fiber and/or protein, improves the GFB quality regarding the GI [21,23,55,56,57]. However, there is a lack of grouped information on the glycemic index of gluten-free bread and the main ingredients used in their formulations to help health professionals and consumers to guide their diet avoiding health impairments. Additionally, we evaluated the methods used in the studies allowing better comprehension of the results found.

### 4.1. Differences between the Used Methods to Evaluate the GI

GI is used to evaluate the nutritional quality of food based on the incremental area under the blood glucose response curve of a 50 g carbohydrate portion of a tested food (expressed as a percentual of the response to the same amount of carbohydrate from a standard food, glucose or white bread taken by the same individual) [40]. 

The primary protocol, defined by the FAO [40], describes only the *in vivo* methodology. However, this method is difficult to reproduce since it depends on the presence of healthy human volunteers, days of repetition, and blood samples. Therefore, the *in vitro* method was created based on the use of enzymatic subtracts to mimic the glycemic response of a food [38,58]. Although this method reproduces reliable results, it is noteworthy that synthetic enzymes and an incubation site may not be able to reproduce the complexity of the human gastrointestinal tract. Uncontrollable varieties such as genetic factors, intestinal length, and synergic interactions between nutrients exert influence on the digestion time and, therefore, on the GI of foods as well [58,59]. In this manner, considering the variability due to the human digestive system, GI’s of foods determined by the *in vitro* method tends to be overestimated when compared to the *in vivo* method [22,58,59,60]. Additionally, it is essential to highlight that some individuals who follow a gluten-free diet present digestive impairments and gut damage, which can also affect their glycemic response to a specific food [50,55].

Methods evaluating the GI applied in the *in vitro* studies of this review were similar, differing in the production of the enzymes and the enzymatic incubation step. The procedure described by Brennan and Tudorica [39], an adaptation of Goñi et al. [38], was the most used one (63.63% of the studies; *n* = 7). It adds a chewing simulation followed by a proteolytic phase and incubation with pancreatic a-amylase in a restricted way, with the use of dialysis tubings, reproducing more reliable GI results [22,24,25,26,30,31]. Two studies used the protocol described only by Goñi et al. [38]. 

The FAO’s protocol describes the differences in the *in vivo* methods between the use of finger-pricking collecting capillary blood and venous blood. Finger-pricking capillary blood is preferred since it is less-invasive, and its results show less variability, making statistical differences between different foods easier to be detected [40]. All the *in vivo* studies included in this review based their methods on the FAO’s protocol, collecting finger-pricking capillary blood samples in five different slots of time within 2 hours [21,23,27,33,37]. However, 60% of the studies which performed *in vivo* analysis (*n* = 3) used glucose as their GI parameter [21,23,33], while the remaining two used white bread [20,27]. Brouns et al. [43] recommend the expression of GI relative to glucose (100). However, for practical purposes, it is accepted to use reference foods other than glucose (such as white bread) during the measurement of GI. This procedure can be conducted as long as it has been calibrated against glucose, and the condition of preparation of this food is standardized [41,43]. According to Wolever et al., [61] if white bread was used as the reference food, the GI values should be multiplied by 0.71 to convert them to the glucose scale (i.e., the GI of glucose = 100). However, the studies [20,32] did not mention if they performed the conversion, potentially limiting the comparison. 

Healthy volunteers from both genders were used in all the studies except for Packer et al. who used type-1 diabetics [37], and Berti et al. [32] who included celiac individuals besides the healthy individual’s group. The FAOs’ protocol advocates that only healthy individuals can participate, since medications involved in glucose metabolism might interfere directly with carbohydrate digestion, and people with gastrointestinal comorbidities, such as celiac disease, may face symptoms since white bread contains gluten [40,62].

Although both *in vivo* and *in vitro* GI are useful and validated tools to evaluate food’s nutritional quality, variations regarding the glycemic response of the same food consumed by different individuals are evident [50]. Subjects’ interindividual characteristics such as anthropometric measures and microbiome reflect on foods GI. Within the official protocol, strict recommendations only allow healthy individuals as subjects, and large parcels of heterogeneous populations are disregarded [50]. Therefore, these important distinctions must be considered to interpret foods’ GI and their influence on overall quality. 

### 4.2. Gluten-Free Bread Samples: GI and Main Ingredients

Nutritional compounds (fat, protein, dietary fiber, antinutrients, organic acids, hydrocolloids, the nature of monosaccharides and starches), and also the cooking processes of food can interfere directly with the GI [40]. Foods that contain fat, protein, and/or fiber affect the overall glycemic response of food by slowing down gastric emptying. These foods slow the digestion of carbohydrates since gastric emptying is a major determinant of postprandial glycemia, attested by the relationship between the blood glucose rise after oral carbohydrate with gastric emptying and the effects of modulation of gastric emptying on postprandial glucose and insulin concentrations) [63]. Additionally, in starch-rich products, the process of starch retrogradation and starch-lipid bindings improve the resistant starch content, which can also reduce their GI [64,65,66]. Therefore, the studies tend to use combinations of different ingredients and processes to improve the nutritional and sensory quality of GFB [21,57,67,68,69,70,71].

**Figure 3:**
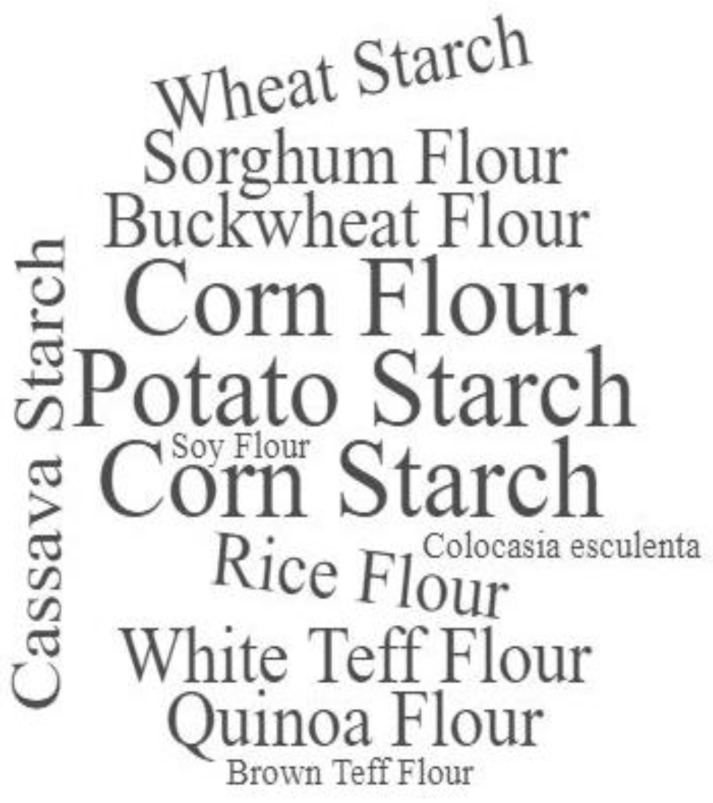
Word Cloud generated from GFB starch sources frequency.

**Figure 4:**
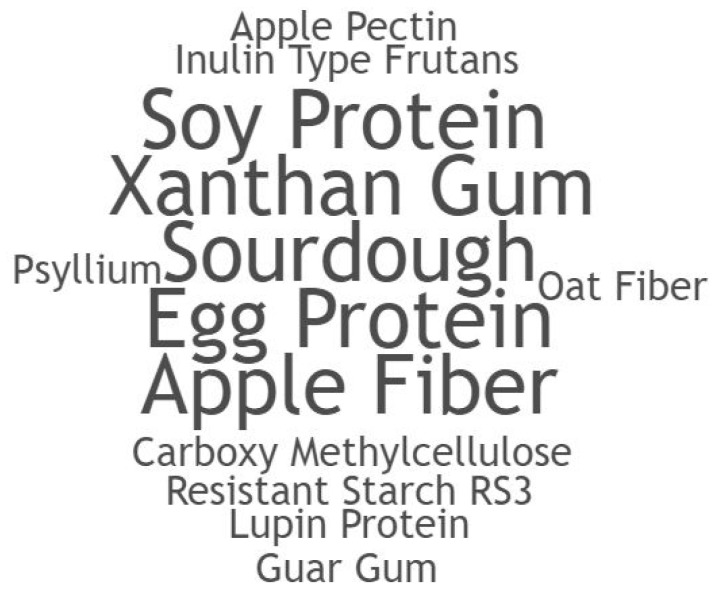
Word Cloud generated from GFB implemented gluten-replacements.

For GF bakery products, to substitute lost technological and sensory characteristics with gluten withdrawal, different starch/flour combinations and enrichment or stabilizing agents are necessary [4,14,71]. Table 2 presents the main starches, stabilizing/enrichment agents in the GFB included in this review, and their respective GI’s. A word cloud generated from implemented starch sources and gluten-replacements is available in the supplementary file (Figure 3 and Figure 4).

Gluten-free starchy ingredients (rice, potato, corn, and cassava) are commonly used in GFB products, usually combined in different proportions. Their rheological characteristics (mainly gelatinization and gelation proprieties) contribute to making GFB with good technological and sensory aspects [21,71,72]. However, since these starches are naturally derived from high GI foods, GFB with these ingredients also tends to present high GIs as well [4]. In the samples included in this systematic review, cornflour and starch, and potato starch were implemented with the highest frequencies (68%) (Figure 3), followed by rice, sorghum, buckwheat, white teff, quinoa, brown teff, and colocasia sculenta flours, respectively. 

Corn, potato, and rice starches typically present high *in vitro* GIs (79, 84, and 86), and in the context of GFB, given that the final volume is majorly composed of starch and these combinations are the most used, GFB may present high GIs despite the use of other ingredients since they are used in small amounts not compromising the sensory quality [41,45,72].

Traditionally, white bread is already classified as a high GI food (89), and dietary approaches generally recommend prioritizing whole grains-based recipes to reduce the GI and improve the nutritional value [41,59,73]. Yet, despite the recent improvements in the nutritional quality of gluten-free products, higher daily GI foods, are still more present on a GFD than non-GFD, given the high GI nature of the implemented main ingredients [55,74,75]. The bread sample that presented the highest GI (99) was composed of cornstarch, rice flour, sugar, vegetable margarine, yeast, hydroxypropyl-methylcellulose, guar gum, salt, lupine proteins, vegetable fiber, and tartaric acid. The GF manufacturer does not inform the amount of sugar, but probably the use of sugar associated with refined high GI starch-ingredients contributed to the very high GI.

Protein and fiber are macromolecules known to mitigate GIs in food preparations in general [75,76], as well as in GFB [77]. However, increased quantities of these ingredients may result in impairments regarding the sensory and technological quality of the GFB [78]. The main explanation is that protein and fiber are components with increased molecular weight and consequently exert pressure in the brittle texture of GFB; therefore, the use of these compounds to lower GFB GI’s is limited [21,31,55,79].

Protein and fat sources such as yogurt and cheese curd were incorporated in GFB made with potato starch and buckwheat and rice flours and successfully decreased the estimated GI [28]. Generally, both ingredients diluted GFB starch granules, and given their elevated molecular weight, exerted influence on carbohydrates digestion, therefore lowering GFB’s GI. Cheese curd, as an ingredient with denser protein and fat content in comparison with yogurt, exerted increased influence and was more successful in lowering the GI. Yet, phenolic compounds present in both ingredients might be able to slow enzyme hydrolysis activity [28].

Pseudocereals, with a higher content of protein and fiber, may be used to obtain lower GIs in GFB. However, high GI starch sources with enhanced capacity to retain water and form gels (such as rice, cassava, and potato) are needed in combination with pseudocereals, since they usually cannot form stable structures, necessary to produce good quality bread [12,23,26,29,35].

Multiple gluten replacers were implemented within the analyzed samples, as shown in Figure 4. Capriles and Arêas [20] used the combination of rice and potato starches with different percentages of inulin-type fructans (ITF), nutritional compounds based on complex carbohydrate chains. They are known to act in a similar way as dietary fiber, forming complex macromolecule structures, slowing digestion, and releasing digested carbohydrates, therefore, lowering the GI [20,22,55]. Although the results with ITF were all classified as a high GI (89, 86, 84, 84), the implementation of ITF reduced the GI (compared with the control sample), progressively lowering the GI with the increase of ITF percentages [20].

Inulin was also used in combination with rice, soy flours, and cassava starch, showing to progressively decrease the GI of GFBs as the percentage of inulin increases [25]. As for the proved health benefits, inulin acts as a prophylactic measure to prevent constipation, a common symptom in GRDs [80]. Its prebiotic potential has also been proven to enhance the absorption of minerals and stimulate the immune system [80,81]. Therefore, given ITFs crescent efficacy in mitigating foods’ GI, its implementation with other ingredients with the same purpose might be useful to improve the glycemic response of GFB.

Potato and rice starches were used in combination with different hydrocolloids in the studies. Segura et al. [35] analyzed GFB brands available in Spain’s local markets. Xanthan and guar gums, carboxymethyl-cellulose (CMC), pectin, and hydroxypropyl-methyl-cellulose (HPMC) were used as gluten replacements and stabilizing agents [29,35,36]. Additionally, in a similar way to dietary fiber, these kinds of hydrocolloids can delay the release of digested carbohydrates and possibly lower the GI of GFB [29,35]. At the same time, hydrocolloids can form denser, slowly digestible molecules in the presence of protein. Hence the bread with milk protein, casein, presented the lowest GI (88) when compared to the other ones analyzed in the study [35]. Different results were found depending on the main starch source implemented. Higher glycemic indexes were found where potato, cassava, and corn starches were used as sources (88,90,83,87,91,91,89,96,89,87). In the case where *Colocasia esculenta* was implemented, combinations with HPMC, xanthan, and guar gums resulted in low GIs (24.58, 23.90, 23.15) [35,36]. However, GFB made with *Colocasia esculenta* showed extremely compact structures, an undesirable sensory characteristic for bread, thus undermining the effectiveness of the measure [36].

Steamed GFB made with fresh potato flour (raw, dehydrated potatoes, processed and sifted) in combination with different hydrocolloids (HPMC, Carboxy Methylcellulose, xanthan gum, and apple pectin) presented medium GIs [29]. Various GIs were found as the concentration of the used hydrocolloid was different (0.5%, 1.0%, 2.0%), with HPMC presenting the lowest GIs (65, 60, and 58 respectively). In contrast, Carboxy-Methylcellulose, xanthan gum, apple pectin showed 66, 68, 66, 62, 63, 65, 64, 65, and 65, respectively for the three different concentrations [29]. Dietary fibers from apples are known to increase total dietary fiber content, therefore, influencing the GI in foods [82]. The cooking process might have also influenced the GFB carbohydrates digestion speed. Hot water steam favors the gelatinization of the starches in the GFB, without at the same time dextrinizing it, thus modifying its bioavailability and consequently lowering the GI [29,38].

Rice flours produced from different cultivars were used as a single starch source. Tarom, Hashemi, Khouzestan, and Lenian are Iranian rice cultivars that differ in their nutritional composition and their content of amylose and amylopectin since their harvests occur in places with contrasting climates. In contrast, the first two occur in mild and humid regions, and the last ones grow in dry places [30]. GFB made with rice flour derived from cultivars from dry places presented medium GIs (66, 64) while the remaining ones presented high GIs (81, 89). Rice from drier places presented higher values of protein and fibers, nutritional compounds directly related to lower GIs. Both can retard the absorption of digested carbohydrates [30,62]. However, it is noteworthy that while GFB made with dry regions’ rice cultivars presented lower GIs and higher protein and fiber values, inferior technological and sensory aspects were also shown [30]. The stabilizing agent used on this GFB formulation might have interfered in the glycemic response as well; however, in this study, this stabilizing agent was not specified [30]. In this sense, this kind of rice cultivar, richer in protein and fiber, when combined with explored hydrocolloids like HPMC and xanthan gum, and mucilage like psyllium, might result in satisfactory GFB, both for sensory and nutritional quality aspects [30].

A study made GFB from an unspecified GF flour mix with the addition of three different types of RS (RS, RS3a, and RS3b) [22]. From the different types of RS, RS3a and 3b refer specifically to the retrograded starch formed with the cooling of gelatinized starch, a rheological phenomenon that occurs within time [22]. Higher contents of RS demonstrated to have a direct correlation with lower GIs, as higher percentages of RS lowers GI values [25]. Considering the health benefits, RS has shown to act similarly as dietary fibers with numerous physiological benefits: it reduces gastric emptying, slowing the digestion process, yet, RS acts as an efficient energy source for colonic probiotic microorganisms (*Bifidobacterium* mostly) capable of producing short-chain fatty acids known to ease intestinal inflammatory processes [83,84]. This functional property is very desirable since GRD individuals usually suffer from recurrent bowel inflammations that may lead to digestive problems with future impairment of nutritional status [85,86]. Additionally, being able to retain water molecules within its structure, RS can likewise improve technological quality in GFB [20]. 

GFB made from pseudocereals presented GIs classified as high by Wolter et al. [24]. Bread samples were prepared with 100% flour (buckwheat, oat, quinoa, sorghum, or teff flours), 2% salt, 2% sugar, and 3% dry yeast. The GFB samples prepared with buckwheat, oat, quinoa, sorghum, and teff flours presented 80, 71, 85, 72, 74 GIs, respectively. Additionally, different fiber, starch, fat, and protein content were found in each GFB sample of the used flours, depending on the type of flour. Quinoa presented lower levels of protein, starch, and fiber; therefore, its digestion is facilitated, resulting in the highest GI of all analyzed samples, followed by the buckwheat flour-based GFB [24]. Teff and sorghum flour naturally present higher amounts of fiber, complex starches, and protein, therefore resulting in slower digestion and, subsequently, a decreased GI compared to the others analyzed in this study [24,62,76].

Oat presented the highest values of fiber and the lowest GI of the analyzed samples (71). Additionally, its fiber in its isolated form was used as enrichment and has shown to decrease GI in other formulations of GFB, reinforcing the direct correlation between higher fiber values and lower GIs [24,25]. Oats can be implemented in a GFD since versions with strict control over cross-contamination with gluten-containing cereals are already available on the market [87,88]

The GFB produced with Psyllium (*Plantago ovata*), rice, and cassava starches, presented a low GI (50) [21]. Psyllium presents multiple health benefits, mainly related to gastrointestinal tract complications, like diarrhea and constipation [71]. Psyllium can be implemented as a strategy to lower the GI of whole meals, being successfully implemented as a tool in obesity treatment [73,89]. As for technological characteristics, psyllium has shown to improve volume, texture, and crumb structure, being very well suited as a gluten replacer in bread [21,71,90,91].

Sourdough is traditionally a yeast replacement based on microorganisms colonies from spontaneous growth [12]. Its implementation in bakery products improves digestibility, the bioavailability of different nutrients, and, in the context of GFB, the products’ palatability [92,93]. Scazzina et al. [33] analyzed the available Italian market sourdough GFB based on rice and millet flours, and rice, corn, and potato starches obtaining low GI (52) [33]. However, since there was no control GFB to compare and the ingredients’ quantities and the presence of stabilizing agents were not specified, there is not enough evidence to directly relate sourdough to the obtained GI [33].

Novotni et al. [23] also utilized sourdough as enrichment for GFB, obtaining low (52, 54) and medium (59, 61) GIs, the last being the one with the higher percentage of implemented sourdough. In their study, higher GIs were found with the introduction of sourdough in GFB. Shumoy et al. [26] implemented sourdough in addition to white and brown teff flours, while Wolter et al. [31] used quinoa, buckwheat, sorghum, and teff flours. Wolter et al. [31] presented mostly high GIs (91.6%), probably because the sourdough microorganisms digest the available starch chains to produce carbonic dioxide responsible for the dough’s growth. The hydrolysis of the starch makes it more digestible, potentially increasing the glycemic response. Additionally, the amount of available mono and disaccharides increases, subsequently raising the GFBs GIs [26,31]. Higher GIs were also proportionally found in GFB with lower levels of protein, fiber, and starch, in a similar way that the other study from the same author has shown [24,31].

Differences between the nature of the microorganisms in the sourdough reflect on the product’s GI. *Weissella cibaria*-based sourdough presented higher GIs than the *Lactobacillus plantarum*-based ones, probably because the first one has a more efficient mechanism for carbohydrate digestion, mainly impacting on the starch hydrolysis [31]. In GFB made with gluten-free wheat starch, the sourdough implemented presented lower GI when compared to various GFBs using different ingredients. Therefore, differences between the other used ingredients in the compared GFB formulations might have influenced this result. The use of stabilizing agents such as vegetal protein, soy protein, milk whey powder, egg albumen, apple fiber, and lupin bean protein, with different starch combinations like quinoa, rice, and tapioca flours exert different influences on the GFB digestion and, therefore, in its GI. It is not possible to directly relate the obtained result to sourdoughs implementation [34].

Storage time directly influenced the GI of GFBs made with white and brown teff flours. As the storage days increase, lower GIs were found, probably because during storage, the starch’s retrogradation makes the carbohydrates less available for digestion (the starch is partially converted to type-3 resistant starch) [26]. 

Another dietary compound that characterizes a variable regarding the GI is fat. In the studies of this review, 61.53% (*n* = 8) [20,21,23,24,25,26,31] utilized vegetable oil as a fat source for making GFB samples; 23.08% (*n* = 3) did not specify the fat source [32,33,37], while the remaining used butter [22] and margarine [35]. In general, fatty acids tend to slower digestion by slowing down intestinal transit [40,94]. Studies demonstrated that different dietary oils/fat induce different postprandial response due to their ability to bind starch granules (depending on fatty acid composition and degree of its unsaturation), resulting in an increased RS content, reduced accessibility for hydrolysis, and higher heat stability of starch-oil complex (type 5 RS) compared to native starch [95,96]. Because of their structure, unsaturated fatty acids, such as vegetable oils, are more susceptible to enzymatic action. They are more digestible, especially compared to saturated and trans fatty acids, as they require more time for thorough digestion [94]. Thus, due to the structural differences between fatty acids used for culinary purposes, unsaturated fatty acids tend to increase GIs, given their facilitated digestibility. However, it is worth noting that carbohydrate structures and bioavailability are still responsible for most of the glycemic response [8,45,97]. 

Although the study did not aim to compare whether the percentages of bread with high GI is statistically higher than those with a low GI, the proportion of bread with a high GI is higher than the low GI ones, with a significance level of 5%, as 95% CI (diamonds) do not intersect. In general, some factors influenced the lower GI gluten-free bread regardless of the starch source such as the use of psyllium [21], and sourdough fermentation [23,33]. The GI of products prepared with teff flour was affected by the storage period, probably because of the starch retrogradation impacting the digestive process [26].

In the studies where a control sample was used, the effects of various gluten replacements on GFB’s GI were evident within the developed products. In Wolter et al. [31], the control GFB samples (developed with buckwheat, quinoa, sorghum, and teff flours, with no hydrocolloids or gluten-replacers, respectively) presented GIs of 80, 95, 72, and 74. In contrast, GFB samples enriched with *Weissella cibaria* presented 89, 106, 81, and 84, and with *Lactobacillus plantarum,* 86, 103, 69, and 68, respectively [32]. Therefore, the authors concluded that the use of *Weissella cibaria* increased the GFB’s GI for all samples, and *Lactobacillus plantarum* was more successful than *Weissella cibaria* in mitigating the GFB GI [32].

A study compared the addition of ITF in a GFB control sample (50% rice flour and 50% potato starch, 25% egg, 10.5% whole milk powder, 6% sugar, 6% soy oil, 2% salt, 0.8% instant dry yeast, 0.3% xanthan gum, 0.3% carboxymethylcellulose and 85% water) [20]. The control sample presented a GI of 93, and the ones with different amounts of ITF presented a gradual GI reduction (4% ITF: 89.98; 8% ITF: 84.88; 10% ITF: 84.97; and 12% ITF: 84.10). Therefore, a reduction of 9.69% in the overall GI was shown with the highest proportion of ITF (12%) in the GFB formula [20]. The addition of 17.14% of psyllium reduced the GI in the GFB sample (GI = 50) by 25.37% compared to the control sample (GI = 67) (composed of 75% rice flour, 25% cassava starch, 25% whole egg, 10.5% whole milk powder, 6% white cane sugar, 6% soy oil, 2% salt and 0.8% dry yeast) [21]. 

A study compared the addition of RS with a GFB control sample (composed of 500 g GF flour mix, 15 g dry yeast, 10 g sugar, 8 g salt, 1.5 g xanthan gum, and 1.5 g carboxymethylcellulose) [22]. The control sample presented a GI of 97, while the one implemented with 20% of RS presented a GI of 88 [22]. Different types of RS were also used (RS3a and RS3b), with GIs of 78 and 70, respectively, resulting in an overall decrease of 20.45% when RS3b is used [22]. 

A study compared the use of oat, inulin, and RS in two different proportions (5% and 10% for the overall yield) to the control GFB sample (composed of 45 g rice flour, 45 g cassava starch, 10 g active soy flour, 2 g salt, 2 g shortening, 3 g compressed yeast and 80 g water) with a GI of 84 [25]. The GFB samples presented GIs of 93 and 71 for the ones enriched with oat; 91 and 81 with inulin, and 81 and 70 with RS, thus, showing RS as the most efficient ingredient to mitigate GFB’s GI [25]. The implementation of oat fiber and inulin at a 5% proportion increased GFB’s GI, whereas, in 10%, the GI decreased. The central hypothesis is that with 5%, the GFB’s protein and starch content was more available for digestion, thus increasing the GI [26]. Additionally, the addition of this specific amount of fiber may have disrupted the GFB crumb structure, therefore favoring the overall digestion and carbohydrate release [26]. In general, higher proportions of ingredient replacements (mainly starches or gluten) tend to result in lower GIs. However, it is important to note that GI results from the synergistic interaction between the added ingredients. Therefore, improvements related to both the choice and the proportion of the starches and the chosen gluten substitutes need to be thoroughly analyzed to obtain a more nutritionally adequate GFB.

Romão et al. [27] compared twelve brands available in the Brazilian nationwide market. Eight were sold as traditional white bread loaves and four as whole-grain versions. In general, a high glycemic index profile was found given that starches with high GIs (cassava and potato starches, rice flour) were implemented in substantial quantities. Medium GI was found in samples commercialized as “whole-grain” options, as pseudocereals, seeds, psyllium, and hydrocolloids were incorporated together in these samples [27]. 

According to the meta-analysis, as expected, there is a moderate/low level of heterogeneity between studies (Figure 2), showing a high proportion of high GIs in GFB. It is important to highlight that the meta-analysis was performed only regarding the GI since only 50% (*n* = 9) of the studies mentioned the amount of the ingredients and the nutritional composition, hindering the analysis of the ingredients [20,21,23,25,27,28,30,34]. In this sense, it was not possible to perform a statistical correlation between GI and ingredients, as a potential limitation of our study.

### 4.3. Glycemic Index Role in GRD

The rising incidence of chronic diseases has become one of the most common causes of death worldwide. Food consumption is also one of the leading causes that increase the risk of developing chronic diseases for people who suffer from a GRD. This risk must be considered more thoroughly in the context of a GRD since GF foods mainly rely on high GI starch sources (since starch is usually hydrolyzed in the human digestive tract into glucose), therefore resulting in high GI final products as well [4,15]. Consuming high glycemic food may cause health problems leading to the increase of obesity, type 2 diabetes, and various non-communicable diseases (NCDs) such as heart failure and cancer [98]. Additionally, overweight or obesity may develop in CD patients after gluten withdrawal. This probably occurs because the mucosal healing following gluten withdrawal is responsible for the overweight increase associated with the ingestion of refined starch-rich GF products [99,100]. The increase in weight gain in patients after dietary gluten exclusion is a potential cause of morbidity, and the gluten-free diet as conventionally prescribed needs to be modified accordingly [100,101].

GRD individuals are most prone to develop nutritional shortcomings because of gastrointestinal health issues and their capacity to digest and absorb nutrients [85,102,103,104]. The advent of GF products with poor nutritional quality, especially regarding micronutrient amounts and high GIs, contributes to raising the risk of these shortcomings in these people [86,102,105].

The development of GF products often faces the challenge of balancing sensory and nutritional quality. Dietary compounds such as refined sugar, different starches, and fat are implemented as an efficient, low-cost approach to replace lost characteristics with gluten withdrawal resulting in satisfactory products [4,106]. However, it is worth highlighting the potential long-term nutritional loss with these replacements. 

The adherence to a life-long strict GF diet is the only safe treatment for all GRD [85,86], and issues regarding availability, cost, and mainly sensory aspects play a significant role in a successful treatment [13,107,108,109]. Refined and high GI white starches such as rice, potato, and cassava are prioritized to produce food with a similar appearance and taste to their gluten-rich counterparts. This practice tends to extend to other GF products as well, resulting in a nutritionally impaired availability of products [4,13,85,106]. Therefore, besides the adherence to a strict GF diet, the nutritional composition and the GI of the available food have to be considered as a tool to evaluate a successful life-long treatment [110].

Celiac disease (CD) and Type-1 Diabetes share the same genetic background since the HLA genotypes DR3-DQ2 and DR4-DQ8 are strongly associated, thus increasing the risk for CD bearers to develop Type-1 diabetes [111]. Additionally, sudden increases in glycemic curves often resulted from the ingestion of high GI foods, which may trigger the early development of diabetes [8,111]. 

Although obesity consists of a multifactorial disease, it is known that a high GI profile of the diet is correlated to rising prevalence, and, in the context of a GRD, the high GI profile of the available products contributes to this increase [8,73,99]. Obesity and overweight among CD individuals are becoming more common due to the increased total energetic value, fat, and sugar content of gluten-free foods, and the nutritional imbalance and hypercaloric content of commercial gluten-free food items [112]. Studies about the influence of a GFD on celiac disease associated with overweight or obesity are necessary to help determine dietary and nutritional interventions. Assessment of existing dietary guidelines and the gluten-free products’ nutritional quality is strongly necessary considering the increasing number of patients with both CD and overweight or obesity [100]. Additionally, high dietary GI significantly increases coronary heart disease risk, and non-favorable effects may be higher in overweight and obese patients [113].

## 5. Conclusions 

Despite the wide variety of starches and flours investigated composing the 116 GFB samples, the studies’ outcomes indicated most of the high GIs for GFB, potentially impacting the development of chronic diseases. Most evaluated GFB samples presented a high GI. However, it was not possible to conclude that all GFB would have a high GI because this would depend much on their formulation. It is necessary to include the consumption of low GI to reduce postprandial glycemia. The use of ingredients with higher contents of dietary fiber and protein and the implementation of resistant starches and fructans have shown to lower GIs in GFB. Despite that, with the evidence of the role of a low GI diet in the prevention of chronic diseases, it is important to highlight the difficulties in the balance of nutritional, technological, and sensory quality on gluten-free products. Further studies are needed better to investigate the long-term effects of regular consumption of GFB.

## Figures and Tables

**Table 1:** Summary of descriptive characteristics and outcomes of interest in the included studies.

Author/Reference	Country	Study Outline	GI Determination Method	Type of Flour/Starch Base	Enrichment	*In vitro*/*In Vivo*	Number of Samples/Subjects Tested
Segura, & Rosell [35]	Spain	Exploratory cross-sectional quantitative study	*In vitro*: Goñi I, Garcia-Alonso A, Saura-Calixto F (1997) [38]	Corn starch and flour, potato starch, rice flour	Soy protein, lupin proteins	*In vitro*	Eleven brands of gluten-free bread from Spain’s market, with duplicates
Wolter et al. [24]	Ireland	Exploratory cross-sectional quantitative study	*In vitro*: Brennan, C.S., amd Tudorica, C.M. (2008). [39]	Buckwheat flour, oat flour, quinoa flour, sorghum flour, teff flour, wheat flour	-	*In vitro*	Six types of bread, with triplicates
Capriles & Arêas [20]	Brazil	Exploratory cross-sectional quantitative study	*In vitro*: Goñi I, Garcia-Alonso A, Saura-Calixto F (1997) [38] *In vivo*: Capillary Blood, FAO/WHO, 1998. [40]	Rice flour, potato starch	Inulin-type fructans	*In vitro* and *In vivo*	Five types of bread, with triplicates One male and nine healthy female subjects
Giuberti et al. [22]	Italy	Exploratory cross-sectional quantitative study	*In vitro*: Goñi I, Garcia-Alonso A, Saura-Calixto F (1997) [38]	Gluten-free flour mix, not specified	Resistant Starch	*In vitro*	Four types of bread, with triplicates
Shumoy et al. [26]	Belgium	Exploratory cross-sectional quantitative study	*In vitro*: Goñi I, Garcia-Alonso A, Saura-Calixto F (1997) [38]	White and brown tef flour	Sourdough	*In vitro*	Four types of bread, with triplicates
Wolter et al. [31]	Ireland	Exploratory cross-sectional quantitative study	*In vitro*: Brennan, C.S., and Tudorica, C.M. (2008). [39]	Buckwheat, flour, quinoa flour, sorghum flour, teff flour, wheat flour	Sourdough	*In vitro*	Five types of bread, with triplicates
Fratelli et al. [21]	Brazil	Exploratory cross-sectional quantitative study	*In vivo*: Capillary Blood, FAO/WHO, 1998. [40] Wolever, T.M.S., Jenkins, D.J.A., (1986). [41]	Rice flour, cassava starch	Psyllium	*In vivo*	Thirteen healthy subjects
Berti et al. [32]	Italy	Exploratory cross-sectional quantitative study	*In vitro*: Brighenti F, Pellegrini N, Casiraghi MC, Testolin G (1995) [42] *In vivo*: Intravenous blood FAO/WHO 1998. [40]	Not specified, brands from the local market	-	*In vitro* and *in vivo*	*In vitro:* two types of conventional bread *In vivo*: Seven healthy female subjects, six celiac female subjects
Feizollahi et al. [30]	Iran	Exploratory cross-sectional quantitative study	*In vitro*: Brennan, C.S., and Tudorica, C.M. (2008). [39]	Rice flour (four varieties of rice), potato flour, corn starch	-	*In vitro*	Four types of bread, with triplicates
Novotni et al. [23]	Croatia	Exploratory cross-sectional quantitative study	*In vivo*: Capillary Blood, FAO/WHO, 1998. [40] Brouns, F., Bjorck, I., Frayn, K.N., Gibbs, A.L., Lang, V., Slama, G., Wolever, T.M.S., (2005). [43]	Rice flour, potato flour, cornflour, buckwheat flour, potato, corn starch.	Sourdough	*In vivo*	Seven female and 4 male healthy subjects
Packer et al. [37]	United Kingdom	Exploratory cross-sectional quantitative study	*In vivo* Intravenous Blood: FAO/WHO, (1998). [40]	Wheat starch	Fiber	*In vivo*	Eleven diabetic (type 2) subjects
Scazzina et al. [33]	Italy	Exploratory cross-sectional quantitative study	*In vivo*: Capillary Blood FAO/WHO (1998) [40]	Rice flour, corn starch, potato starch, millet flour, rice starch	Soy protein, apple fiber, lupin protein, Sourdough	*In vivo*	Ten male and ten female healthy subjects
Sciarini et al. [25]	Argentina	Exploratory cross-sectional quantitative study	*In vitro*: Goñi I, Garcia-Alonso A, Saura-Calixto F (1997) [38]	Rice flour, cassava starch, soy flour	Resistant starch RS3, oat fiber and inulin	*In vitro*	Three types of bread, with triplicates
Rizzello et al. [34]	Italy	Exploratory cross-sectional quantitative study	*In vitro*: Brennan, C.S., and Tudorica, C.M. (2008). [39]	Wheat starch without gluten; millet flour	Wheat sourdough without gluten 50% (water, durum wheat flour, lactic acid bacteria);	*In vitro*	One gluten-free bread.
Liu et al. [29]	China	Exploratory cross-sectional quantitative study	*In vitro*: Dartois, A, Singh J., Kaur L. Singh H. (2010). [44]	Fresh potato flour	Hydroxypropyl-MethylCelullose, CarboxymethylCellulose, Xanthan Gum, Apple Purée	*In vitro*	Twelve types of bread.
Calle et al. [36]	Spain	Exploratory cross-sectional quantitative study	*In vitro*: Goñi I, Garcia-Alonso A, Saura-Calixto F (1997) [38]	*Colocasia esculenta* flour	HPMC, Xanthan Gum, Guar Gum, Gluzyme Mono 10.000 BG, iZyme BA	*In vitro*	Five Types of Bread
Romão et al. [27]	Brazil	Exploratory cross-sectional quantitative study	*In vivo*: Capillary Blood, FAO/WHO, 1998. [40] Brouns, F., Bjorck, I., Frayn, K.N., Gibbs, A.L., Lang, V., Slama, G., Wolever, T.M.S., (2005). [43]	Cassava and potato starches and rice flour	HPMC, Xanthan Gum, Guar Gum, Psyllium, Soy Protein, Lupin Protein, Apple Fiber	*In Vivo*	Twelve Types of Bread
Graça et al. [28]	Portugal	Exploratory cross-sectional quantitative study	*In vitro*: Goñi I, Garcia-Alonso A, Saura-Calixto F (1997) [38]	*Buckwheat, potato starch, and rice flour*	Yogurt and Cheese Curd	*In vitro*	Four Types of Bread

**Table 2:** Main ingredients and Glycemic Indexes of gluten-free bread (GFB) presented in the studies.

Study	Starch Sources	Stabilizing Agent/Enrichment Ingredient	GI	GI Classification [45]
Giuberti et al. [22]	Not Specified	None	97	High
		RS 20%	88	High
		RS3a 20%	78	High
		RS3b 20%	70	High
Berti et al. [32]	Not Specified	Not Specified	230	High
Scazzina et al. [33]	Rice flour, corn starch, potato starch, millet flour, rice starch	Sourdough	52	Low
		Soy Protein	62	Medium
		Apple Fiber	63	Medium
Capriles and Arêas [20]	Rice flour, potato starch	None	93	High
		4% ITF	89	High
		8% ITF	86	High
		10% ITF	84	High
		12% ITF	84	High
Fratelli et al. [21]	Rice flour, cassava starch	None	67	Medium
		Psyllium	50	Low
Wolter et al. [24]	Buckwheat Flour	None	80	High
	Oat Flour		71	High
	Quinoa Flour		85	High
	Sorghum Flour		72	High
	Teff Flour		74	High
Wolter et al. [31]	Buckwheat Flour	Control	80	High
		WC	89	High
		LP	86	High
	Quinoa Flour	Control	95	High
		WC	106	High
		LP	103	High
	Sorghum Flour	Control	72	High
		WC	81	High
		LP	69	Medium
	Teff Flour	Control	74	High
		WC	84	High
		LP	78	High
Segura et al. [35]	Corn Starch	Xanthan Gum	87	High
		Xanthan Gum, guar gum, pectin, CMC	90	High
		Guar gum, pectin, CMC	83	High
	Potato starch, corn starch	Casein, soy protein, HPMC, xanthan gum	87	High
	Corn Starch, rice flour	Guar gum, HPMC, lupine protein, vegetal fiber	91	High
	Corn Starch	Xantham Gum	91	High
		Xantham Gum, HPMC	91	High
		Xantham Gum	89	High
		Xantham Gum	96	High
		Xantham Gum	89	High
		Xantham Gum	88	High
Shumoy et al. [26]	White Teff Flour	Sourdough		
		Fresh: 0%, 10%, 20%, 30%	72, 82, 77, 86	High
		1 day: 0%, 10%, 20%, 30%	58, 67, 62, 54	Low, Medium, Medium, Low
		2 days: 0%, 10%, 20%, 30%	51, 55, 62, 60	Low, Low, Medium, Medium
		5 days: 0%, 10%, 20%, 30%	39, 50, 45, 52	Low, Low, Low, Low
	Brown Teff Flour	Fresh: 0%, 10%, 20%, 30%	75, 83, 85, 89	High, High, High, High
		1 day: 0%, 10%, 20%, 30%	72, 70, 74, 74	High, High, High, High
		2 days: 0%, 10%, 20%, 30%	66, 69, 74, 74	Medium, Medium, High, High
		5 days: 0%, 10%, 20%, 30%	66, 69, 74, 73	Medium, Medium, High, High
Feizollahi et al. [30]	Tarom rice flour	Non specified stabilizers	81	High
	Hashemi rice flour		89	High
	Khouzestan rice flour		66	Medium
	Lenian rice flour		64	Medium
Novotni et al. [23]	Rice flour, potato flour, cornflour, buckwheat flour, potato purée, corn starch	7.5 g Sourdough	59	Low
		15 g sourdough	52	Low
		22.5 g sourdough	54	Low
		30 g sourdough	61	Medium
Packer and Frost [37]	GF wheat starch commercial unsliced white bread	None	101	High
	GF wheat starch commercial sliced white bread		114	High
	GF wheat starch commercial sliced fiber-enriched bread	Unspecified fiber	99	High
	GF wheat starch commercial fiber-enriched white bread		109	High
Sciarini et al. [25]	Rice flour, cassava starch, soy flour	None	84	High
		5% Oat fiber	93	High
		10% Oat fiber	71	High
		5% Inulin	91	High
		10% Inulin	81	High
		5% Resistant Starch	81	High
		10% Resistant Starch	70	High
Rizzello et al. [34].	Wheat starch without gluten; millet flour	Wheat sourdough without gluten 50% (water, durum wheat flour, lactic acid bacteria);	74	High
Liu et al. [29]	Fresh potato flour	0.5% HPMC	65.02	Medium
		1% HPMC	60.52	Medium
		2% HPMC	58.89	Medium
		0.5% CMC	66.25	Medium
		1% CMC	68.38	Medium
		2% CMC	66.57	Medium
		0.5% XG	62.71	Medium
		1% XG	62.70	Medium
		2.0% XG	63.28	Medium
		0.5% AP	65.09	Medium
		1.5% AP	64.83	Medium
		2.0% AP	65.12	Medium
Calle et al. [36]	*Colocasia esculenta* flour	HPMC	24.58	Low
		Xanthan Gum	23.90	Low
		Guar Gum	23.15	Low
		Gluzyme Mono 10.000 BG	26.20	Low
		iZyme BA	26.32	Low
		Alcalase 1.5 MG Type FG	23.10	Low
		Potato Starch	32.81	Low
Romão et al. [27]	*Cassava and potato starches and rice flour (Local Market samples)*	GFB 1	67.97	Medium
		GFB 2	64.00	Medium
		GFB 3	70.14	High
		GFB 4	78.72	High
		GFB 5	77.69	High
		GFB 6	79.94	High
		GFB 7	76.53	High
		GFB 8	75.39	High
		WGFB 1	67.66	Medium
		WGFB 2	61.46	Medium
		WGFB 3	69.23	Medium
		WGFB 4	75.40	High
Graça et al. [28]	*Gluten-free flour mix: Buckwheat, potato starch, and rice flour*	YgB 10%	82.80	High
		YgB 20%	76.50	High
		CcB 10%	68.00	Medium
		CcB 20%	62.70	Medium

RS: Resistant Starch; ITF: Inulin-Type Frutans; WC: Weissella cibaria; LP: Lactobacillus Plantarum; CMC: CarboxyMethylCellulose; HPMC: HydroxyPropylMethylCellulose; XG: Xanthan Gum; GFB: Gluten-free Bread; WGFB: Whole-grain Gluten Free Bread; YgB: Yogurt Bread; CcB: Cheese curd.

## Data Availability

The study did not report any data.

## Supplementary Materials

The following are available online at https://www.mdpi.com/2304-8158/10/3/506/s1, Table S1. Quality criteria of the selected studies for the systematic review of the gluten-free bread glycemic index; Table S2—Risk of Bias of the Included Studies; Table S3—Search Strategy.
