# Supplementary material for: Glycemic Index of Gluten-Free Bread and Their Main Ingredients: A Systematic Review and Meta-Analysis
Source: Foods. 2021 Feb 27;10(3):506. doi: 10.3390/foods10030506 (PMC7996770; doi:10.3390/foods10030506)
Supplement: Supplementary file 1 [file foods-10-00506-s001.pdf]

S1. Systematic Review Protocol

METHODS

Question: If applicable use the PICO acronym as a guide.

**Glycemic index of gluten-free bread and their main ingredients**

**PICOs**	
**Participants / food/ beverages**	**Human subjects or in vitro human digestion simulation**
**Intervention or exposition**	**Glycemic Index Gauging**
**Comparison or control**	**Glucose or White Bread**
**Outcome measure(s)**	**Glycemic impact**
**Types of Studies included**	**Scientific articles**

Eligibility criteria

Inclusion criteria **Specify the study characteristics (e.g., PICO, study design, setting, time frame) and report characteristics (e.g., years considered, language, publication status) to be used as criteria for eligibility for the review**

- **Studies *in vitro* using starch hydrolysis and predicted glycemic index of gluten-free bread**
- **Studies *in vivo* using glycemic curves and index on humans to determine the glycemic impact of gluten-free bread**
- **Studies comparing the impact of different ingredients and respective contents in the glycemic index of gluten-free bread**

Exclusion criteria: **Do not list the negative of the inclusion criteria. List here additional criteria.**

1- Reviews, letters, conference abstracts, and books

2- Other studies

Information sources: Describe all intended information sources (e.g., electronic databases, contact with study authors, trial registers, or other grey literature sources) with planned dates of coverage.

Electronic searches up to will be conducted using the following electronic bibliographic databases: PubMed, Scopus, Lilacs. The initial search strategy will be designed for PubMed and adapted to other databases. After the study selection, we will search the reference list of included articles. In addition, we will search grey literature (google scholar, ProQuest and experts suggestions) to include other articles that were not included previously.

Databases:

1. (x) PubMed

2. (x) EMBASE

3. (   ) Cochrane

4. (   ) Medline

5. ( ) LILACS

6. (x) Web of Science

7. (x) Science Direct

8. ( ) PsycINFO

10. (X) Other: Ovid; wiley of library medline, springerlink; scopus;

Additional Literature:

1. (x) Google Scholar web search (specify if limitations applied)

2. (x) Hand searches of bibliographies from included studies

3. (x) Experts

4. ( ) Proquest (Dissertation and Theses)

Others: Opengrey

Search strategy: Present draft of search strategy to be used for all the electronic database searches, including planned limits, such that it could be repeated.

Database	Search
**Pubmed**	***("glycaemic index"[All Fields] OR "glycemic index"[All Fields] OR "glycemic impact"[All Fields] OR "glycaemic impact"[All Fields] OR "glycemic index"[MeSH Terms] OR ("glycemic index"[MeSH Terms] OR ("glycemic"[All Fields] AND "index"[All Fields]) OR "glycemic index"[All Fields] OR ("glycemic"[All Fields] AND "index"[All Fields] AND "number"[All Fields])) OR ("glycemic index"[MeSH Terms] OR ("glycemic"[All Fields] AND "index"[All Fields]) OR "glycemic index"[All Fields] OR ("glycemic"[All Fields] AND "index"[All Fields] AND "numbers"[All Fields])) OR "Glycemic Indices"[All Fields] OR "Blood glucose"[All Fields] OR "blood sugar"[All Fields] OR "glycemic response"[All Fields] OR "glycemic responses"[All Fields] OR "glycaemic response"[All Fields] OR "glycaemic responses"[All Fields] OR "postprandial glycemia"[All Fields] OR "postprandial blood glucose response"[All Fields] OR "postprandial blood glucose responses"[All Fields] OR "postprandial blood glucose"[All Fields] OR "postprandial glucose"[All Fields] OR "glycemic curve"[All Fields] OR "Hydrolysis curve"[All Fields] OR "starch hydrolysis"[All Fields] OR "starch digestion"[All Fields] OR "starch absorption"[All Fields] OR "nutritional qualities"[All Fields] OR "nutritional quality"[All Fields] OR***

	"nutritional balance"[All Fields]) AND ("gluten-free diet"[All Fields] OR "gluten free"[All Fields] OR "diet, gluten-free"[MeSH Terms] OR "gluten-free"[All Fields] OR "gluten-free products"[All Fields] OR "gluten free products"[All Fields])
Embase	("gluten-free diet" OR "gluten-free" OR "gluten-free" OR "gluten-free products" OR "gluten-free products") AND ("glycaemic index" OR "glycemic index" OR "glycemic impact" OR "glycaemic impact" OR "glycemic index" OR "Glycemic Index Number" OR "Glycemic Index Numbers" OR "Glycemic Indices" OR "Blood glucose" OR "blood sugar" OR "glycemic response" OR "glycemic responses" OR "glycaemic response" OR "glycaemic responses" OR "postprandial glycemia" OR "postprandial blood glucose response"OR "postprandial blood glucose responses" OR "postprandial blood glucose" OR "postprandial glucose" OR "glycemic curve" OR "Hydrolysis curve" OR "starch hydrolysis" OR "starch digestion" OR "starch absorption" OR "nutritional qualities" OR "nutritional quality" OR "nutritional balance"

Data management: Describe the mechanism(s) that will be used to manage records and data throughout the review.

1. (x) Endnote

2. (   ) Refworks

3. (x) Mendeley

4. (x) Other: Rayyan

Selection process: State the process that will be used for selecting studies (e.g., two independent reviewers) through each phase of the review (i.e., screening, eligibility, and inclusion in meta-analysis).

**Two reviewers independently will review the list of titles and abstracts for inclusion according to the inclusion criteria. The full articles selected for phase II will be read by two reviewers independently to decide whether these meet the inclusion criteria. If there is disagreement in screening and eligibility phases, a third reviewer will be contacted to make the decision.**

Data collection process: Describe the planned method of extracting data from reports (e.g., piloting forms, done independently, in duplicate), any processes for obtaining and confirming data from investigators.

**Data will be extracted by two reviewers independently on the following characteristics: author and year of publication, sample, ingredients, nutritional quality, the method used to identify the glycemic impact, and other variables, findings, and conclusions. To ensure consistency across reviewers, we will conduct calibration exercises before starting the review. Reviewers will resolve disagreements by discussion, and the third reviewer will adjudicate unresolved disagreements.**

Risk of bias assessment: Describe anticipated methods for assessing the risk of bias of individual studies, including whether this will be done at the outcome or study level, or both; state how this information will be used in data synthesis. If more than one study type will be included then more than one risk of bias assessment tool may be required.

**We will use the specific GRADE critical appraisal tools for assessing the risk of bias in a comparable cohort, case-control, and cross-sectional studies. The two reviewers will conduct a quality assessment of the study. Reviewers will resolve disagreements by discussion, and the third reviewer will adjudicate unresolved disagreements. The quality of the studies will be described in the table and figure.**

Data Synthesis: Describe criteria under which study data will be quantitatively synthesized. If data are appropriate for quantitative synthesis, describe planned summary measures, methods for data handling and combination, including any planned exploration of consistency (e.g., I^2). Describe any proposed additional analyses (e.g., sensitivity or subgroup analyses, meta-regression). If quantitative synthesis is not appropriate, describe the type of summary planned.

**The characteristics of interest of the studies will be described in the table as informed in "Data collection process". If there are a sufficient number of comparable studies with a common outcome and exposure measurement, a meta-analysis can be used to combine statistical evidence from more than one study to estimate a pooled effect.**

<u>Analysis of subgroups or subsets</u>: Describe any planned quantitative exploration of subgroups or subsets within the review. 'None planned' is a valid response if no subgroup analyses are planned.

**1. glycemic index measured in vivo**<br>**2.glycemic index measured in vitro**

Type of review:

1.(   ) Therapeutic (intervention)

2.(   ) Diagnosis

3.(   ) Risk factors

4.( ) Epidemiologic (frequency, prevalence)

5.(X) Other

Previous systematic reviews about the same subject: ( ) yes (x) no

Suggest 3 journals in which this research could be published and why:

**Critical reviews in food science**

**Nutrition**
**Food Reviews International**

Cite 5 studies that you read (about this topic) before preparing this protocol:

**Reference 1: Conte P, Fadda C, Piga A, Collar C. Techno-functional and nutritional performance of commercial bread available in Europe. Revista de Agaroquimica y Tecnologia de Alimentos. 2016 Oct;22(7):621-33.**
**Reference 2: Berti C, Riso P, Monti LD, Porrini M. In vitro starch digestibility and in vivo glucose response of gluten-free foods and their gluten counterparts. European Journal of Nutrition. 2004 Aug 1;43(4):198-204.**
**Reference 3: Segura ME, Rosell CM. Chemical composition and starch digestibility of different gluten-free breads. Plant Foods for Human Nutrition. 2011 Sep 1;66(3):224.**
**Reference 4: Capriles VD, Arêas JA. Approaches to reduce the glycemic response of gluten-free products: in vivo and in vitro studies. Food & function. 2016;7(3):1266-72.**
**Reference 5: Capriles VD, Arêas JA. Approaches to reduce the glycemic response of gluten-free products: in vivo and in vitro studies. Food & function. 2016;7(3):1266-72.**

Table S1. Quality criteria of the selected studies for the systematic review of the gluten-free bread glycemic index.

Reference	Were the analyzed products characterized?	Was the method of analysis specified?	Was the method used certified/validated by Codex and/or AOAC?	Was the result of glycemic index determined quantitatively?	Were the methods of consumption of gluten-free bread or sample homogenization of the study samples described?	Was the experimental design appropriate?	Was the statistical adequate to the purpose of the study?	Did the results answer the main question?	Were the blood glucose responses/glycemic index well described?	Percentage of positive answers (yes) for each study that attained the quality criteria	Risk of bias
Berti et al. (2004)	N	Y	N	Y	Y	Y	Y	Y	N	66.66%	Moderate
Capriles et al. (2013)	Y	Y	N	Y	Y	Y	Y	Y	Y	88.88%%	Low
Feizollahi et al. (2018)	Y	Y	Y	Y	Y	Y	Y	Y	Y	100%	Low
Fratelli et al. (2018)	Y	Y	NA	N	Y	Y	Y	Y	N	75%	Low
Giuberti et al. (2016)	N	Y	Y	Y	N	Y	Y	Y	Y	77.77%	Low
Novotni et al. (2012)	Y	Y	NA	Y	Y	Y	Y	Y	Y	100%	Low
Packer et al. (2000)	Y	Y	NA	Y	Y	Y	Y	Y	Y	100%	Low
Scazzina et al. (2015)	Y	Y	NA	Y	Y	Y	Y	Y	Y	100%	Low
Sciarini et al. (2017)	Y	Y	Y	Y	N	Y	Y	Y	Y	88.88%	Low
Segura et al. (2011)	Y	Y	Y	Y	Y	Y	Y	Y	Y	100%	Low

Shumoy et al. (2018)	Y	Y	N	Y	N	Y	Y	Y	Y	77.77%	Low
Wolter et al. (2013)	Y	Y	Y	Y	N	Y	Y	Y	Y	88.88%	Low
Wolter et al. (2014)	Y	Y	Y	Y	N	Y	Y	Y	Y	88.88%	Low
Rizello et al. (2016)	Y	Y	Y	Y	N	Y	Y	Y	Y	88,8%	Low
Liu et al. (2017)	Y	Y	Y	Y	N	Y	Y	Y	Y	88,8%	Low
Calle et al., (2019)	Y	Y	Y	Y	N	Y	Y	Y	Y	88,8%	Low
Romão et al. (2020)	Y	Y	Y	Y	Y	Y	Y	Y	Y	100%	Low
Graça et al. (2019)	Y	Y	Y	Y	Y	Y	Y	Y	Y	100%	Low

Legend: Y = Yes; N = No; NA= Not Applicable

Table S2 – Risk of Bias of the Included Studies

Author, year	Risk of bias	Risk percentage
Giuberti, G. et al., (2016)	Low	77.77%
Berti, C. et al., (2004)	Moderate	66.66%
Scazzina, F. et al., (2015)	Low	100%
Capriles, V. D.; Arêas, J. A. (2013).	Low	88.88%%
Fratelli, C. et al., (2018)	Low	75%
Wolter, A. et al., (2013)	Low	88.88%
Wolter, A. et al., (2014).	Low	88.88%
Segura, M. E. M.; Rosell, C. M. (2011).	Low	100%
Shumoy, H. et al., (2018).	Low	77.77%
Feizollahi, E. et al., (2018).	Low	100%
Novotni, D. et al., (2012).	Low	100%
Packer, S. C. et al., (2000).	Low	100%
Sciarini, L. S. et al., (2017).	Low	88.88%

Rizello et al., (2016)	Low	88.88%
Liu et al., (2017)	Low	88.88%
Calle et al (2019)	Low	88.88%
Romão et al (2020)	Low	100%
Graça et al (2019)	Low	100%

Table S3 - Search Strategy

Database	Search (February 1st, 2020)
PUBMED	*("glycaemic index"[All Fields] OR "glycemic index"[All Fields] OR "glycemic impact"[All Fields] OR "glycaemic impact"[All Fields] OR "glycemic index"[MeSH Terms] OR ("glycemic index"[MeSH Terms] OR ("glycemic"[All Fields] AND "index"[All Fields]) OR "glycemic index"[All Fields] OR ("glycemic"[All Fields] AND "index"[All Fields] AND "number"[All Fields])) OR ("glycemic index"[MeSH Terms] OR ("glycemic"[All Fields] AND "index"[All Fields]) OR "glycemic index"[All Fields] OR ("glycemic"[All Fields] AND "index"[All Fields] AND "numbers"[All Fields])) OR "Glycemic Indices"[All Fields] OR "Blood glucose"[All Fields] OR "blood sugar"[All Fields] OR "glycemic response"[All Fields] OR "glycemic responses"[All Fields] OR "glycaemic response"[All Fields] OR "glycaemic responses"[All Fields] OR "postprandial glycemia"[All Fields] OR "postprandial blood glucose response"[All Fields] OR "postprandial blood glucose responses"[All Fields] OR "postprandial blood glucose"[All Fields] OR "postprandial glucose"[All Fields] OR "glycemic curve"[All Fields] OR "Hydrolysis curve"[All Fields] OR "starch hydrolysis"[All Fields] OR "starch digestion"[All Fields] OR "starch absorption"[All Fields] OR "nutritional qualities"[All Fields] OR "nutritional quality"[All Fields] OR "nutritional balance"[All Fields]) AND ("gluten-free diet"[All Fields] OR "gluten free"[All Fields] OR "diet, gluten-free"[MeSH Terms] OR "gluten-free"[All Fields] OR "gluten-free products"[All Fields] OR "gluten free products"[All Fields])*
Science Direct	("gluten free" OR "gluten-free") AND ("glycaemic index" OR "glycemic index" OR "glycemic response" OR "glycaemic response" OR "glycemic curve" OR "blood sugar" OR "blood glucose" OR "nutritional quality" OR "starch absorption")
Scopus	(TITLE-ABS-KEY ("gluten-free diet" OR "gluten free" OR "gluten-free" OR "gluten-free products" OR "gluten free products")) AND ((TITLE-ABS-KEY ("postprandial glucose" OR "glycemic curve" OR "Hydrolysis curve" OR "starch hydrolysis" OR "starch digestion" OR "starch absorption" OR "nutritional qualities" OR "nutritional quality" OR "nutritional balance")) OR (TITLE-ABS-KEY ("glycemic index" OR "glycemic index" OR "glycemic impact" OR "glycemic impact" OR "glycemic index" OR "Glycemic Index Number" OR "Glycemic Index Numbers" OR "Glycemic Indices" OR "Blood glucose" OR "blood sugar" OR "glycemic response")) OR (TITLE-ABS-KEY ("glycemic responses" OR "glycemic response" OR "glycemic responses" OR "postprandial glycemia" OR "postprandial blood glucose response" OR "postprandial blood glucose responses" OR "postprandial blood glucose"))

Web of Science	TS=("gluten-free diet" OR "gluten free" OR "gluten-free" OR "gluten-free products" OR "gluten free products") AND ((TS=("glycaemic index" OR "glycemic index" OR "glycemic impact" OR "glycaemic impact" OR "glycemic index"[MeSH Terms] OR "Glycemic Index Number" OR "Glycemic Index Numbers" OR "Glycemic Indices" OR "Blood glucose" OR "blood sugar" OR "glycemic response" OR "glycemic responses" OR "glycaemic response" OR "glycaemic responses" OR "postprandial glycemia" OR "postprandial blood glucose response"OR "postprandial blood glucose responses" OR "postprandial blood glucose" OR "postprandial glucose" OR "glycemic curve" OR "Hydrolysis curve" OR "starch hydrolysis" OR "starch digestion" OR "starch absorption" OR "nutritional qualities" OR "nutritional quality" OR "nutritional balance"))
EMBASE	(('gluten-free diet'/exp OR 'gluten-free diet' OR 'gluten free' OR 'gluten-free' OR 'gluten-free products' OR 'gluten free products') AND ('glycaemic index' OR 'glycemic impact' OR 'glycaemic impact' OR 'glycemic index'/exp OR 'glycemic index' OR 'glycemic index number' OR 'glycemic index numbers' OR 'glycemic indices' OR 'blood glucose'/exp OR 'blood glucose' OR 'blood sugar'/exp OR 'blood sugar' OR 'glycemic response'/exp OR 'glycemic response' OR 'glycemic responses' OR 'glycaemic response' OR 'glycaemic responses' OR 'postprandial glycemia'/exp OR 'postprandial glycemia' OR 'postprandial blood glucose response' OR 'postprandial blood glucose responses' OR 'postprandial blood glucose'/exp OR 'postprandial blood glucose' OR 'postprandial glucose' OR 'glycemic curve' OR 'hydrolysis curve' OR 'starch hydrolysis'/exp OR 'starch hydrolysis' OR 'starch digestion'/exp OR 'starch digestion' OR 'starch absorption' OR 'nutritional qualities' OR 'nutritional quality'/exp OR 'nutritional quality' OR 'nutritional balance'/exp OR 'nutritional balance'))
Google Scholar	"glycemic index" "gluten-free bread"
Google Scholar	"gluten free bread" "blood glucose response"
